# ConnecTF: A platform to integrate transcription factor–gene interactions and validate regulatory networks

**DOI:** 10.1093/plphys/kiaa012

**Published:** 2020-11-18

**Authors:** Matthew D Brooks, Che-Lun Juang, Manpreet Singh Katari, José M Alvarez, Angelo Pasquino, Hung-Jui Shih, Ji Huang, Carly Shanks, Jacopo Cirrone, Gloria M Coruzzi

**Affiliations:** 1 Center for Genomics and Systems Biology, Department of Biology, New York University, NY, USA; 2 USDA ARS Global Change and Photosynthesis Research Unit, Urbana, IL, USA; 3 Centro de Genómica y Bioinformática, Facultad de Ciencias, Universidad Mayor, Santiago, Chile; 4 Millennium Institute for Integrative Biology (iBio), Santiago, Chile; 5 Courant Institute for Mathematical Sciences, Department of Computer Science, New York University NY, USA

## Abstract

Deciphering gene regulatory networks (GRNs) is both a promise and challenge of systems biology. The promise lies in identifying key transcription factors (TFs) that enable an organism to react to changes in its environment. The challenge lies in validating GRNs that involve hundreds of TFs with hundreds of thousands of interactions with their genome-wide targets experimentally determined by high-throughput sequencing. To address this challenge, we developed ConnecTF, a species-independent, web-based platform that integrates genome-wide studies of TF–target binding, TF–target regulation, and other TF-centric omic datasets and uses these to build and refine validated or inferred GRNs. We demonstrate the functionality of ConnecTF by showing how integration within and across TF–target datasets uncovers biological insights. Case study 1 uses integration of TF–target gene regulation and binding datasets to uncover TF mode-of-action and identify potential TF partners for 14 TFs in abscisic acid signaling. Case study 2 demonstrates how genome-wide TF–target data and automated functions in ConnecTF are used in precision/recall analysis and pruning of an inferred GRN for nitrogen signaling. Case study 3 uses ConnecTF to chart a network path from NLP7, a master TF in nitrogen signaling, to direct secondary TF_2_s and to its indirect targets in a Network Walking approach. The public version of ConnecTF (https://ConnecTF.org) contains 3,738,278 TF–target interactions for 423 TFs in Arabidopsis, 839,210 TF–target interactions for 139 TFs in maize (*Zea mays*), and 293,094 TF–target interactions for 26 TFs in rice (*Oryza sativa*). The database and tools in ConnecTF will advance the exploration of GRNs in plant systems biology applications for model and crop species.

## Teaser

ConnecTF is a web application/database enabling users to build and validate gene regulatory networks by combining transcription factor–target binding and regulation datasets for model plants and crops.

## Introduction

Deciphering gene regulatory networks (GRN) is an important task, as it can reveal regulatory loci, like transcription factors (TFs), that are crucial for development, stress responses, or disease, with potential applications in agriculture and medicine (Petricka et al., 2012; Chatterjee and Ahituv, 2017; Gupta and Singh, 2019). However, integrating experimentally validated connections between TFs and their genome-wide target genes in such GRNs remains a challenge. 

With the advent of next-generation sequencing, there are a growing number of methods to validate TF–target gene connections within GRNs, each with its own set of benefits and drawbacks. Methods that provide evidence for where a TF is likely to bind to the genome include: chromatin immunoprecipitation sequencing (ChIP-seq), DNA affinity purification sequencing (DAP-seq; O'Malley et al., 2016), and cis-motif enrichment. To determine when TF-binding leads to target gene regulation requires the integration of TF-binding data with TF-regulation datasets. However, large-scale datasets that validate TF–target gene regulation data are sparse relative to the TF–target gene binding data themselves. This is largely due to the low-throughput nature of TF-perturbation approaches in planta (e.g. overexpression or mutants). Thus, there is a need for high-throughput methods to rapidly identify direct regulated TF–targets in plants. One such method is the transient assay reporting genome-wide effects of transcription factors (TARGET), which identifies direct regulated TF–targets in isolated plant cells based on changes in target gene expression after temporally controlled TF nuclear entry, as reported for Arabidopsis (Bargmann et al., 2013; Brooks et al., 2019). Protoplasts have also recently been used in a high-throughput assay to identify ChIP-seq data for 103 TFs performed in isolated maize (*Zea mays*) cells (Tu et al., 2020).

Such large-scale datasets for TF–target gene binding or regulation can be used to verify predictions of TF–target gene connections in GRNs (Marbach et al., 2012; Banf and Rhee, 2017; Mochida et al., 2018; Kulkarni and Vandepoele, 2019). Validated TF–target interactions can also be used as priors (e.g. “ground truths”) to train machine learning in network inference methods (Greenfield et al., 2013; Petralia et al., 2015; Cirrone et al., 2020), and/or as a gold standard with which to benchmark/refine the accuracy of predicted TF–target interactions in learned GRNs (e.g. using precision/recall analysis; Marbach et al., 2012; Varala et al., 2018; Brooks et al., 2019). We have also previously shown that the integration of TF–target binding with TF–target regulation datasets can be used to discover distinct modes-of-action of a TF on induced vs. repressed gene targets (Brooks et al., 2019).

Platforms that facilitate access to and integration of such large-scale datasets that validate TF–target gene interactions are crucial to accelerate studies of validated and inferred GRNs. To this end, there are efforts to aggregate TF–target datasets, largely comprising TF-binding and cis-motif elements, for many species, including human (Han et al., 2018), yeast (Monteiro et al., 2019), *E. coli* (Santos-Zavaleta et al., 2019), and Arabidopsis (Yilmaz et al., 2010; Kulkarni et al., 2018; Tian et al., 2019). There are also web portals that provide access to specific experimental datasets that support TF–target binding, for example, the Plant Cistrome database for large-scale assays of in vitro TF–target binding (DAP-seq; O'Malley et al., 2016). Primarily, these platforms allow users to query a TF and obtain a list of TF-bound target genes or vice versa.

Despite these advances, few, if any, current platforms enable a combined analysis of TF-bound genes, TF-regulated genes, and co-expression data, or the ability to combine such datasets to refine/validate predicted GRNs. An important feature that is missing from most available web tools is the ability to integrate genome-wide targets of a single TF validated by different experimental approaches (e.g. ChIP-seq, DAP-Seq, and RNA-seq), captured under the same or different experimental conditions. A second feature that is currently lacking is the ability to compare the validated targets of multiple TFs and determine their hierarchy in a GRN, as they relate to a set of user-defined genes such as a pathway of interest. Finally, tools are also needed to facilitate the refinement/pruning of predicted GRNs by using the validated TF–target interactions from genomic studies to perform precision/recall analysis.

To meet the need in the systems biology community to build, validate, and refine GRNs, we developed ConnecTF, a platform that offers a query interface to access a TF-centric database consisting of large-scale validated TF–target gene interactions based on TF–target binding (e.g. ChIP/DAP-Seq) and other gene-to-gene directed (e.g. TF–target regulation) or undirected (e.g. TF–TF protein–protein interaction) relationships. We are hosting a publicly available instance of ConnecTF (https://ConnecTF.org), which includes a database of large-scale validated TF–target interactions containing TF-binding (in vivo and in vitro), TF-regulation (in planta and in plant cells), and cis-motif datasets for the model plant Arabidopsis and the crops, maize and rice (*Oryza sativa*). The ConnecTF database currently contains 3,738,278 experimentally validated TF–target interactions for 423 TFs in Arabidopsis (Table 1), 839,210 experimentally validated TF–target interactions for 139 TFs in maize (Supplemental Table S1), and 293,094 TF–target interactions for 26 TFs in rice (Supplemental Table S1). The ConnecTF database also includes the largest TF–target regulation dataset in plants, specifically, the direct regulated targets for 58 TFs in Arabidopsis (Varala et al., 2018; Brooks et al., 2019; Alvarez et al., 2020; this study).

**Table 1:** Overview of the validated Arabidopsis TF–target datasets in the ConnecTF database. For overviews of maize and rice TF datasets in ConnecTF, see Supplemental Table S1

Interaction type	Experiment type	No. of TFs	No. of edges	Reference
TF-binding	ChIP-seq	26	257,400	(Song et al., 2016; Birkenbihl et al., 2017)
	DAP-seq	382	3,335,595	(O'Malley et al., 2016)

TF-regulation	in planta perturbation	3	7,894	(Marchive et al., 2013; Varala et al., 2018)
	TARGET (plant cells)	58	137,389	(Brooks et al., 2019; Alvarez et al., 2020; this study)

TF–TF protein–protein interactions	HaloTag-NAPPA CrY2H	1,221	6,555	(Yazaki et al., 2016; Trigg et al., 2017)
cis-binding motifs	TF cis-binding motifs	1,310 cis-motifs for 730 TFs collected from Cis-BP		(Weirauch et al., 2014)
	cis-Motif clusters	80 clusters from 1,282 individual cis-binding motifs		(Brooks et al., 2019)

**Figure 1:**
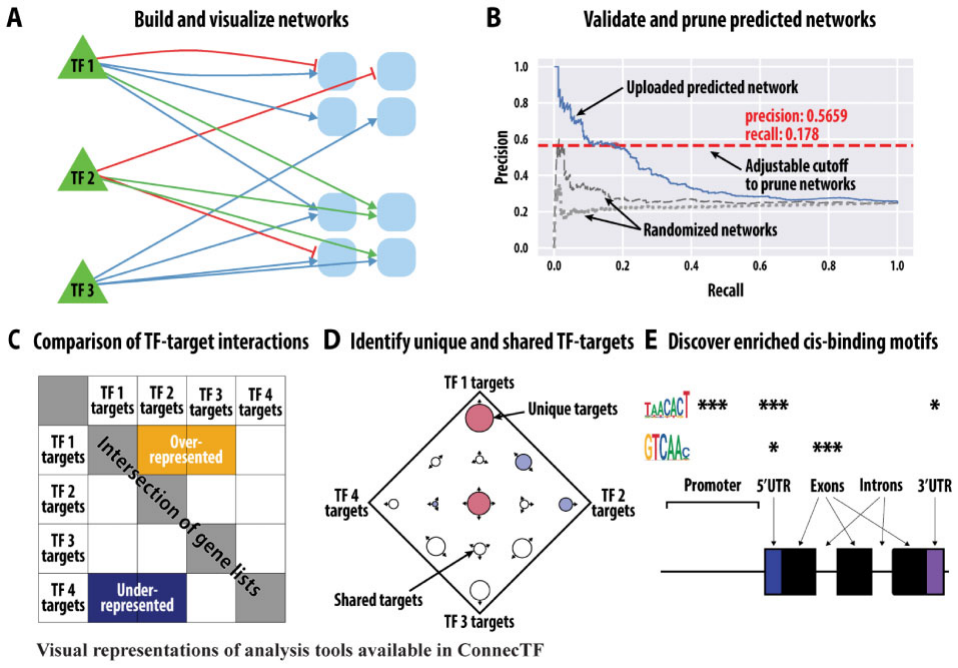
Representations of the analysis and visualization tools in ConnecTF for the integration of data supporting TF–target gene interactions to build/validate gene regulatory networks. ConnecTF contains TF–target interactions for 707 experiments from Arabidopsis, 158 experiments in maize, and 63 experiments in rice, for a total of 4.87 million TF–target interactions for 616 TFs (Table 1 and Supplemental Table S1). The distinct types of validated TF–target data within each species can be filtered and integrated using analysis/visualization tools within ConnecTF to: A, build and visualize validated gene regulatory networks; B, use validated TF–target data to perform precision/recall analysis and prune predicted networks (user uploaded or predefined in database); C, compare whether the TF–targets in common between two experiments/TFs are overrepresented or underrepresented; D, determine how TF–targets are distributed between TF experiments; and E, identify enriched cis-binding motifs in validated TF targets.

We demonstrate in three case studies how the features of ConnecTF (Figure 1) and its ability to integrate a large and diverse variety of validated TF–target gene datasets can provide biological insights into GRNs. In the first case study, we demonstrate how the integration of validated TF-binding and TF-regulation datasets enabled us to discover how TFs and their TF–TF partner interactions influence the regulation of genes in the abscisic acid (ABA) pathway. In case study 2, we demonstrate how ConnecTF can be used to facilitate precision/recall analysis of inferred nitrogen regulatory networks using gold-standard validated TF–target interactions stored in the ConnecTF database. In case study 3, we demonstrate how the query system of ConnecTF can be used to integrate validated TF–target datasets from multiple TFs into a unified network path. Specifically, using the query functions in ConnecTF, we were able to chart a network path from the direct targets of NIN-LIKE PROTEIN 7 (NLP7), a key TF in the nitrogen response (Marchive et al., 2013; Alvarez et al., 2020), to its indirect targets in planta, using an adaptation of an approach developed in Brooks et al. (2019) called Network Walking. Overall, the database and analysis/integration tools of ConnecTF can be used to advance the validation of GRNs involved in any pathway using systems biology approaches in model or crop species.

## Results

### ConnecTF: A query interface and database to integrate TF–target gene interactions of different data types

The ConnecTF platform enables researchers to readily access, integrate, and analyze a database of experimentally validated TF–target gene interaction datasets via a web interface. The types of TF–target interactions housed in the ConnecTF database can include TF-binding, TF-regulation, TF–TF protein interactions, and cis-motifs. In addition to accessing the data that we are currently hosting, users can create an independent instance of ConnecTF that contains any dataset of their choice. An important feature of ConnecTF is that, in addition to providing researchers access to the large-scale validated TF–target datasets housed in the database, it also offers a user-friendly interface to perform analyses to combine these various datasets for one or many TFs. This includes the ability for users to provide their own target gene list(s) or predicted network and identify the TFs that regulate their pathway/network of interest. Users can also provide their own inferred networks and use the validated TF–target data in the ConnecTF database as a gold standard to perform precision/recall analysis using automated functions. These applications are described in the three case studies below.

The backend structure and tools available in ConnecTF are species-independent and built using common software (Supplemental Figure S1). The source code and detailed instructions on how to setup a personalized version of ConnecTF are available on GitHub (https://github.com/coruzzilab/connectf_server). This will enable researchers to set up their own instance of ConnecTF for private use or public sharing of any TF-centric genomic data. We are hosting public versions of ConnecTF populated with TF–target validation datasets from Arabidopsis (https://ConnecTF.org/), maize (https://Maize.ConnecTF.org/), or rice (https://Rice.ConnecTF.org/). The current version of the Arabidopsis ConnecTF database primarily houses TF-binding or TF-regulation datasets that have been performed at scale (Table 1), enabling direct comparisons of TF–target gene interactions. The Arabidopsis datasets currently in ConnecTF include: 388 TFs for which TF–target binding was identified in vitro by DAP-seq (O'Malley et al., 2016), 21 TF–target binding datasets identified in planta by ChIP-seq (Song et al., 2016), and 58 TFs for which direct regulated TF–target genes were identified in isolated plant cells (Varala et al., 2018; Brooks et al., 2019; Alvarez et al., 2020), including 14 TFs from our current study (Supplemental Table S2). For maize, the ConnecTF datasets include the recently reported ChIP-seq data for 103 TFs performed in isolated maize cells (Tu et al., 2020), TF perturbation and ChIP binding datasets collected from the literature (Bolduc et al., 2012; Morohashi et al., 2012; Eveland et al., 2014; Li et al., 2015), as well as in vitro TF–target binding identified by DAP-seq for 32 maize TFs (Ricci et al., 2019; Supplemental Table S1). As there are no large-scale datasets for rice, in planta TF-perturbation and ChIP binding data was collected as reported from the literature, or raw reads were reanalyzed when necessary (see “Materials and Methods”; Supplemental Table S1). Finally, for both Arabidopsis and maize, we have also included in the ConnecTF database ATAC-seq (Lu et al., 2019) and DNA hypersensitivity (DHS; Sullivan et al., 2014) datasets, which enable users to filter TF–target interactions (e.g. TF–target gene binding) for those occurring in open chromatin regions of the different tissues from those studies.

A key feature of ConnecTF is its logic-based query system. A query in ConnecTF is built by constructing a series of constraints to restrict the set of TFs, the set of target genes, the type of interaction (e.g. TF–target edge type), or other attributes associated with the data. The result of a query is the network (or subnetwork) of interactions for the selected set of TFs and their targets. This query system allows users to select a single TF or multiple TFs of interest, filter the TF–targets based on different criteria (e.g. regulation by a signal of interest, e.g. ABA), and integrate validated TF–target data across multiple TFs. This includes the ability to search for targets of all TFs in the database, or a selected subset of TFs of interest. The query system also allows users to perform analyses based on the experimental type of validated TF–target interaction (e.g. TF-binding) or any other criteria in the metadata (e.g. TF–target assays performed in leaf vs. root). Queries can be built using the graphical *Query Builder* interface or by typing queries into the search text box. This makes the query system easy to use both for researchers new to the ConnecTF site, and for those who wish to build complex queries to parse multiple types of experimentally verified TF–target gene datasets for the TFs available in the database.

ConnecTF also includes several analysis and visualization tools for data integration (Figure 1), whose utility we demonstrate in three case studies. Once a query has been submitted and is processed, the *Summary* tab is loaded and gives an overview of the total number of validated TF–target genes for each experiment that was queried, grouped by individual TFs. The validated TF–target interactions are then made available in the *Table* tab, which provides an interactive table that can be downloaded for offline use in either Excel or CSV formats. The five remaining tabs in ConnecTF allow users to analyze the queried data in various ways (Figure 1): (1) *Network* tab; provides access to a TF–target gene network that can be visualized using Cytoscape.js (Franz et al., 2015; Figure 1A) or downloaded as a JSON or SIF file, (2) *Target List Enrichment* tab; displays the overlap between a user-submitted gene list(s) and the validated TF–targets bound and/or regulated by the queried TF(s) and calculates statistical enrichment, (3) *Motif Enrichment* tab; performs statistical tests for cis-motif enrichment in the validated targets of queried TFs (Figure 1E), (4) *Gene Set Enrichment* tab; calculates the significance of overlap (either greater or less than expected) between the validated targets of each TF analysis, when compared pairwise (Figure 1C), and (5) *Sungear* tab; compares the significance of overlaps between TF–targets from multiple gene lists, comparable to a Venn diagram, but better suited to analyze more than three lists (Figure 1D; Poultney et al., 2006). The *Network* tab also enables users to upload a predicted network and use validated TF–target datasets housed in the ConnecTF database to perform an automated precision/recall analysis. This function generates an area under precision recall (AUPR) curve with an interactive sliding-window feature that can be used to select a precision cutoff with which to prune/refine the predicted network (Figure 1B; Marchive et al., 2013; Banf and Rhee, 2017; Varala et al., 2018; Brooks et al., 2019). The three case studies below provide examples for the use of ConnecTF to investigate TF-function in GRNs by combining each of these features.

### Getting started: Basic queries in ConnecTF

The most basic query in ConnecTF is to enter a TF name/symbol or Gene ID, which will return all of the experiments in the database that validate the TF–target gene interactions for that specific TF. To demonstrate, we submitted a query for NLP7 (AT4G24020), a master regulator in the nitrogen signaling pathway (Marchive et al., 2013; Alvarez et al., 2020), and the results returned from the ConnecTF database included seven experiments for NLP7: four ChIP-seq experiments performed in isolated root cells (Alvarez et al., 2020), one in vitro TF–target binding experiment using DAP-seq (O'Malley et al., 2016), one TF overexpression experiment that identifies direct regulated targets of NLP7 in isolated root cells (Alvarez et al., 2020), and one experiment identifying NLP7-regulated targets based on the analysis of an *nlp7* mutant in planta (Marchive et al., 2013). These results can be viewed in the *Table* tab on the ConnecTF site or downloaded as an Excel file (Supplemental Table S3), and list the validated NLP7 target genes from any one of these experiments. This list includes descriptions of the validated NLP7 target genes (where available) and other details such as edge count (e.g. number of experiments where an interaction between the TF and this target are validated), *P*-value and log2 fold change, if available.

Determining the validated TF–target genes within a pathway or network of interest for one TF, or a set of TFs, is another common task that can be readily performed using ConnecTF. When a query is submitted in ConnecTF, the user can limit the target gene set to one or more lists of genes using the *Target Gene List* box located below the *Query Builder*. We demonstrate this feature using the same NLP7 query as above, but in this example, from the *Target Gene List* box, we select the predefined list of time-dependent nitrogen-response genes obtained from shoot or root (Varala et al., 2018) named “Nitrogen_by_Time”. By selecting this N-by-Time gene list, the validated targets of NLP7 retrieved from the ConnecTF database are now restricted to the genes that are in one of these two pre-defined sets of genes responsive to N as a function of time (in roots or shoots). In the results *Table* tab for this query, there are two additional columns that indicate each gene list (e.g. N-by-Time responsive in roots or shoots), to which the validated NLP7 targets belong (Supplemental Table S3). Uploading a *Target Gene List* also allows the user to determine the enrichment of gene targets of the TF in that pathway viewed in the *Target List Enrichment* tab.

### Case study 1: Uncovering mechanisms of TF mode-of-action and TF–TF interactions by integrating TF–target binding, TF–target regulation, and cis-element datasets

In case study 1, we demonstrate how to use the query functions and data housed in ConnecTF to integrate TF–target gene regulation and TF–target binding data to elucidate the TF mode-of-action, including its potential TF partners. In our previous study of 33 TFs, we showed that by integrating TF-binding and TF-regulation data, we could discover that a single TF can either induce *or* repress target genes (Brooks et al., 2019). Specifically, we showed examples where direct TF–target binding (e.g. via cis-motif enrichment and DAP-seq binding) was associated with TF-mediated target gene induction, whereas indirect binding of the same TF via TF partner(s) (e.g. only captured by ChIP) could account for TF-mediated repression of a target gene, or vice versa (Brooks et al., 2019). However, we were unable to generalize such TF mode-of-action discoveries, as only 3/33 TFs in that prior study had both in vitro and in vivo TF–target binding data to compare to the TF–target regulation data. To expand our discoveries of whether these distinct TF modes-of-action could be generalized, we used ConnecTF to integrate new TF-regulation data we generated in this study (Supplemental Table S2) with existing TF-binding data (Song et al., 2016) for 14 TFs in the ABA signaling pathway. We did this by using functions in ConnecTF to integrate: (i) the direct regulated TF targets of these 14 TFs identified in root cells (Supplemental Table S2) using the TARGET system (Bargmann et al., 2013; Brooks et al., 2019), (ii) in planta TF-binding (e.g. ChIP-seq; Song et al., 2016), (iii) at least one cis-binding motif available on Cis-BP (Weirauch et al., 2014), and (iv) validated in vitro TF-binding data obtained by DAP-seq (O’Malley et al., 2016) for 5/14 of the ABA-responsive TFs.

#### Validated targets of 14 TFs are specifically enriched in ABA-responsive genes

**Figure 2:**
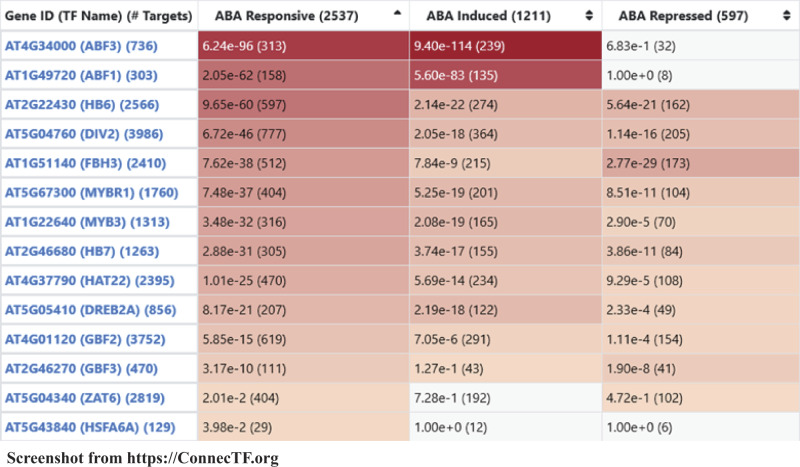
Case study 1: Ranking significance of 14 TFs in regulation of ABA-responsive genes. Screenshot from the ConnecTF website demonstrates how the *Target List Enrichment* tool can be used to address whether the direct regulated targets of 14 ABA-responsive TFs identified in isolated root cells using the TARGET assay (Supplemental Table S2) are enriched for ABA-responsive genes identified in planta by Song et al. (2016). The validated regulated targets of each of the 14 TFs are enriched for ABA-responsive genes, including either ABA-induced genes or ABA-repressed genes (*P* < 0.05, Fisher’s exact test). Known ABA regulators ABF1 and ABF3 (Choi et al., 2000) are among the most enriched in ABA-responsive genes. *Query*: all_expression, *Target Genes*: Abscisic_Acid_Responsive, *Filter TFs*: ABA TFs, *Background*: TARGET_Expressed.

First, we demonstrate how the validated TF–target gene datasets for these 14 ABA-responsive TFs housed in the ConnecTF database can be integrated to understand how they regulate ABA signaling. To do this, we first used the *Target List Enrichment* tool in ConnecTF to determine for each of the 14 TFs whether the validated TF-regulated target genes identified by controlled TF-nuclear import in root cells using the TARGET assay (Bargmann et al., 2013; Brooks et al., 2019) were significantly enriched in a list of ABA-responsive genes identified in planta from Song et al. (2016). This integrated analysis showed that the direct regulated targets of these 14 TFs in isolated plant cells are each significantly enriched for ABA-responsive genes in planta (Fisher’s exact test, *P* < 0.05; Figure 2). This analysis enabled us to address whether each of the 14 TFs are involved in regulating genes that are induced or repressed in response to ABA treatment (Figure 2). Moreover, this analysis revealed that two known regulators of ABA signaling, ABF1 and ABF3 (Choi et al., 2000), are at the top of the list of TFs (ranked by *P*-value) for having targets that are highly enriched for the ABA-induced genes (Figure 2). Next, we further separated the TF-regulated targets of each of the 14 TFs into TF-induced or -repressed target sets using the *Query* function of ConnecTF. This is possible because TF–target gene interaction datasets in ConnecTF that are based on expression optionally include *P*-value and log2 fold-change information for each interaction. This information allows users to separate TF-induced and -repressed targets from the same experiment by building a query that specifies log2 fold-change (e.g. AT2G46680[log2fc > 0] for induced TF–target genes). This analysis enabled us to determine the TF–target *specificity* (e.g. percentage of TF-regulated targets that are ABA responsive), TF–target *influence* (e.g. percentage of ABA-responsive genes regulated by each TF), and *P*-value of the overlap of TF–target genes with induced and repressed ABA-responsive genes (Supplemental Table S4). This analysis revealed that for the majority of the 14 TFs, the TF-induced targets overlap significantly with genes induced by the ABA signal, whereas TF-repressed targets overlap significantly with the genes repressed by ABA treatment.

#### Distinct cis-motifs are enriched in TF-induced and/or TF-repressed targets of 14 TFs in ABA signaling

We next sought to use the TF–target gene binding and TF–target gene regulation data for these 14 TFs to determine whether the TFs act alone, or in combination, to regulate the target genes in the ABA response pathway. To this end, we first asked whether the validated cis-binding motif for each TF (collected from Cis-BP; Weirauch et al., 2014) showed specific enrichment exclusively in either the TF-induced or the TF-repressed target gene lists, as we found in a previous study of 33 TFs in the nitrogen-response pathway (Brooks et al., 2019). To do this, we first made a query in ConnecTF that returns the TF-induced or TF-repressed targets for each TF as separate gene lists. Next, we selected the *Individual Motifs* tab from within the *Motif Enrichment* results page. The default setting returns the cis-element enrichment in the 500-bp promoter region of the validated target genes of a TF for any cis-motif for that TF. Users can also define/select other genic regions of target genes (2,000-bp promoter, 1,000-bp promoter, 5’ untranslated region (UTR), coding sequence (CDS), introns, 3’ UTR, and exons), or choose a cis-motif for another TF, e.g. a putative partner, and ConnecTF will calculate enrichment for the selected motif(s) in the selected genic region(s).

**Figure 3:**
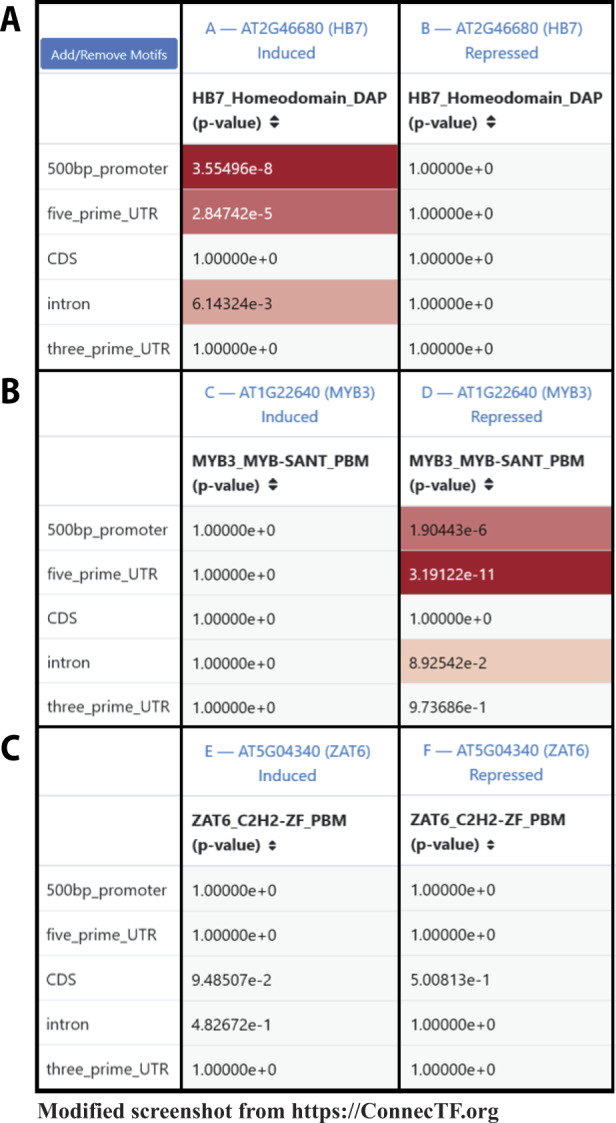
Case study 1: Known cis-binding motifs for a TF are enriched in specific subsets of TF-regulated genes (induced *or* repressed). A screenshot demonstrating how ConnecTF can be used to determine the enrichment of cis-motifs within the subset of targets of a TF (e.g. TF-induced or TF-repressed targets). The ConnecTF database houses 1,310 experimentally determined cis-binding motifs for 730 Arabidopsis TFs, 17 cis-binding motifs for 12 maize TFs, and 26 cis-binding motifs for 23 rice TFs (Table 1 and Supplemental Table S1). Users can use this resource to determine if any of these cis-motifs are enriched in the targets of the queried TF(s) using the *Individual Motifs* section of the *Motif Enrichment* tab. The results show that: A, the HB7 cis-motif is enriched only in the list of TF–target genes induced by HB7 in a root cell-based TF-perturbation assay TARGET, but not in the list of target genes whose expression is repressed by HB7; B, the MYB3 cis-motif is enriched only in the list of TF–target genes repressed by MYB3, but not the list of MYB3-induced target genes; and C, the known motif for ZAT6 is not found to be enriched the list of genes whose expression is either induced or repressed by ZAT6 perturbation. *P*-values were calculated using the Fisher’s exact test. *Query*: AT2G46680[log2fc < 0] or AT2G46680[log2fc > 0] or AT1G22640[log2fc < 0] or AT1G22640[log2fc > 0] or AT5G04340[log2fc < 0] or AT5G04340[log2fc > 0], *Background*: TARGET_Expressed.

For the 14 TFs in the ABA pathway, we examined their TF-induced vs. TF-repressed gene target lists for enrichment of their own cis-motif and show examples for the TFs HB7, MYB3, and ZAT6, (Figure 3). We found that the majority of the 14 TFs tested have enrichment of their known cis-element in *either* their induced *or* repressed targets that we identified as directly regulated TF–targets in root cells using the TARGET assay (Supplemental Table S5). Of these, 7/14 TFs (including HB7, Figure 3A) show enrichment of at least one known cis-motif for that TF exclusively in the TF-induced targets, whereas 2/14 (MYBR1 and MYB3, Figure 3B) show specific enrichment of cis-motif for that TF exclusively in the TF-repressed targets (Supplemental Table S5). For 5/14 TFs (including ZAT6, Figure 3C), there was no enrichment of their known cis-binding motif in either the TF-induced or -repressed targets.

Whereas cis-motif enrichment indicates where a TF is *likely* to directly bind in the genome, validated direct binding to specific genomic loci is available from in vitro TF–target gene binding (e.g. DAP-seq experiments) housed in the ConnecTF database (O'Malley et al., 2016). For the 5/14 ABA-responsive TFs for which DAP-seq data is available (FBH3, GBF3, HB6, HB7, and MYBR1), our comparison of TF-induced or -repressed targets with in vitro TF-bound targets supported the cis-motif enrichment results. That is, for FBH3, HB7, and HB6, only the TF-induced target gene lists were enriched for genes that were bound in vitro to that TF, whereas for MYBR1, only TF-repressed targets were enriched in genes that were bound in vitro to that TF (Supplemental Table S6). GBF3, which had no cis-motif enrichment in either the TF-induced or -repressed directly regulated targets, also had no enrichment of TF-binding in vitro in either set of TF-regulated targets (Supplemental Table S6).

#### TF-regulated genes are largely TF bound, whereas the majority of TF-bound genes are infrequently TF-regulated

**Figure 4:**
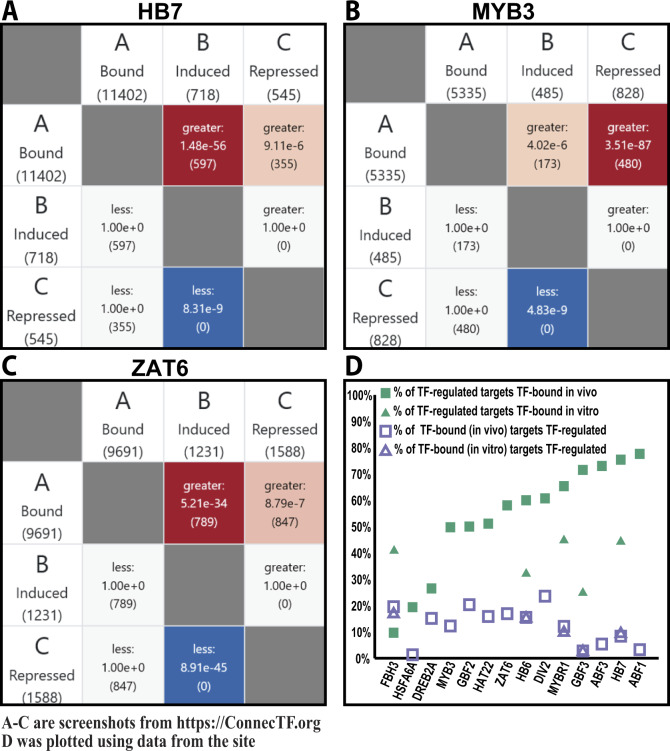
Case study 1: TF-regulated gene targets are largely TF-bound, while TF-bound genes are infrequently TF-regulated. The *Gene Set Enrichment* tool in ConnecTF can be used to determine if the pairwise overlap of the target gene lists of two TF analyses is significant (Fisher’s exact test). This feature enables users to answer common questions such as “When does TF binding lead to TF-regulation (e.g. significance of overlap of TF-binding and TF-regulation)? Or, how significant is the overlap of the list of gene targets of two different TFs?” We demonstrate this feature using three examples: A, HB7, B, MYB3, and C, ZAT6. We display screenshots from the ConnecTF site of the overlap between TF-bound targets, as determined by in planta ChIP (Song et al., 2016) and the TF-regulated targets (e.g. induced or repressed) that we determined in isolated root cells using the TARGET assay (Supplemental Table S2). For each TF, the TF-bound targets significantly overlap with the lists of both the TF-induced and TF-repressed gene targets identified in root cells using the TARGET assay. D, Overlap of TF-regulation and TF-binding for all 14 TFs (Supplemental Table S6 and Supplemental Table S7). Here, we observed that the percent of TF-regulated genes that are TF bound is much greater than the percent of TF-bound genes that are TF-regulated, regardless of whether the binding data is in vivo or in vitro. This suggests that TF-binding alone is a poor indicator of gene regulation in the absence of complementary TF-regulation data for each TF. *Example Query (Panel A):* AT2G46680[log2fc < 0] or AT2G46680[log2fc > 0] or AT2G46680[EDGE_TYPE="in planta:Bound"], *Background:* TARGET_Expressed.

An outstanding question related to TF–target validation datasets is when and whether TF-binding leads to gene regulation. To conduct this analysis, we asked whether genes that are bound by each of the 14 ABA-responsive TFs in planta, based on ChIP-seq experiments (Song et al., 2016), significantly overlap with TF-regulated genes (e.g. either TF-induced or -repressed) identified in root cells using the TARGET assay (Supplemental Table S2). To do this, we used the *Gene Set Enrichment* tool in ConnecTF, which reports whether the pairwise overlap between any two queried experimental analyses is greater or less than that expected by chance (Fisher’s exact test). This *Gene Set Enrichment* function is based on the Genesect tool in VirtualPlant (Katari et al., 2010) and is fully described in Krouk et al. (2010). As an example, for three TFs—HB7, MYB3, and ZAT6—the *Gene Set Enrichment* results show that both the TF-induced and -repressed target gene lists significantly overlap with the TF-bound targets of that TF (*P* < 0.05, Fisher’s exact test; Figure 4, A–C). Extending this analysis to all 14 ABA-responsive TFs, we find that 9/14 TFs have a significant overlap of TF-bound genes in planta with *both* the list of TF-induced *and* -repressed targets for that TF, as validated in root cells using the TARGET assay (*P* < 0.05, Fisher’s exact test; Supplemental Table S7). For 4/14 of the TFs—ABF1, ABF3, DREB2A, and HSFA6A—we found a significant overlap of the TF-bound targets *only* with the TF-induced targets (*P* < 0.05, Fisher’s exact test). By contrast, only 1/16 TFs (GBF2) had a significant overlap of TF-bound targets *only* with the list of TF-regulated targets that are repressed (*P* < 0.05, Fisher’s exact test; Supplemental Table S7).

Importantly, when we used ConnecTF to evaluate the relationship of TF-regulation vs. TF-binding datasets, our integrated analysis showed that for 11/14 of the ABA-responsive TFs, greater than 50%, and as much as 75%, of TF–target genes that were TF regulated in root cells were also bound by that TF in planta (Figure 4D, solid green squares). By contrast, for all 14 TFs, the number of TF-bound targets in planta that were regulated by that TF never exceeded 25% (Figure 4D, open purple squares).

#### Enabling the identification of partner TF cis-binding motifs in focus TF-regulated genes

We next used ConnecTF to identify potential TF partners for each TF being studied, which we refer to as the “focus TF”, a term used in the ENCODE and maize TF-binding network studies (Gerstein et al., 2012; Tu et al., 2020). This was especially relevant when the validated focus TF-regulated targets (either induced or repressed) showed no enrichment of the known cis-binding motif for the focus TF (Supplemental Table S5). In these sets of focus TF-regulated genes, we used ConnecTF to search for overrepresentation of cis-motifs for potential partner TFs. To stream-line this analysis, rather than searching for all 1,310 cis-motifs available for Arabidopsis from Cis-BP (Weirauch et al., 2014), we limited our search to the 80 cis-motif clusters generated from all available Arabidopsis thaliana cis-motifs (Brooks et al., 2019), which are now housed in the ConnecTF database.

**Figure 5:**
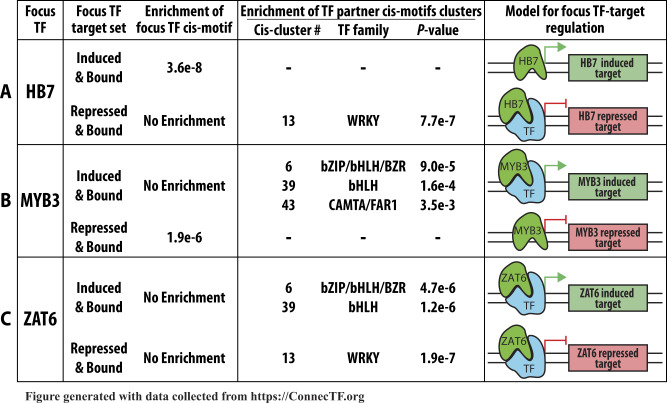
Case study 1: cis-motifs for putative TF partners are identified in indirectly bound focus TF–targets. ConnecTF was used to combine the new TF-regulation data generated in root cells using TARGET assay (Supplemental Table S2), for 14 ABA-responsive TFs with existing TF-binding data in planta (Song et al., 2016). The combination of these datasets reveals the mode-of-action for how these focus TFs function to regulate target genes in the ABA signaling pathway. Here, we summarize these results for 3/14 focus TFs: A, HB7, B, MYB3, and C, ZAT6. For both of the focus TFs, HB7 and ZAT6, we found that the TF-repressed and TF-bound targets, which lack enrichment of the known cis-motif for these focus TF (see Figure 3), had enrichment of the cis-motif cluster (Brooks et al., 2019) representing potential partners in the WRKY TF family. Similarly, for the focus TFs MYB3 and ZAT6, the TF-induced and TF-bound targets that were not enriched in the cis-motif for these focus TFs, were each enriched for cis-motif clusters 6 and 39 (Brooks et al., 2019), which represents potential partner TFs in bZIP/bHLH/BZR families of TFs. This cis-analysis allowed us to derive a model for each focus TF (e.g. HB7, MYB3, and ZAT6), which describes how physical interactions with putative partner TFs enable the focus TF to regulate subsets of its target genes, even in the absence of direct binding. *Example Query (Panel A)*: (AT2G46680[log2fc > 0] and AT2G46680[EDGE_TYPE="in planta:Bound"]) or (AT2G46680[log2fc < 0] and AT2G46680[EDGE_TYPE="in planta:Bound"]), *Background*: TARGET_Expressed.

First, we performed cis-motif enrichment analysis on the validated target gene lists of three focus TFs, namely HB7, MYB3, and ZAT6 (Figure 5). For each of these focus TFs, we hypothesized that they could act directly on gene targets, or through TF partners, based on our analysis of TF-regulation, TF-binding, and cis-motif enrichment. For the focus TF HB7, whereas both induced and repressed targets of HB7 identified in root cells overlap significantly with genes bound by HB7 in planta (by ChIP-seq; Figure 4A), the known HB7 cis-motif is only enriched in the HB7-induced targets (Figure 3A). Using cis-analysis functions in ConnecTF, we found that the HB7-repressed target gene list is specifically enriched in a cis-motif (cis-cluster 13) for WRKY TFs (*P* < 0.05, Fisher’s exact test; Figure 5A). This finding suggests HB7 repression of gene targets is mediated by one or more TF partners in the WRKY TF family. For the focus TF MYB3, whereas both induced and repressed targets of MYB3 identified in root cells are each enriched in genes bound by MYB3 in planta (e.g. ChIP-seq; Figure 4B), the MYB3 cis-motif is only enriched in the list of MYB3-repressed targets (Figure 3B). By contrast, the list of MYB3-induced targets are enriched in cis-motifs (cis-clusters 6, 39, 68) for potential TF partners in the bZIP/bHLH/BZR and CAMTA/FAR1 TF families (*P* < 0.05, Fisher’s exact test; Figure 5B). This result suggests that MYB3 induces target genes via an indirect interaction with partner TF(s) from the bZIP1/bHLH/BZR or CAMTA/FAR1 families. Lastly, although the list of induced and repressed targets of the focus TF ZAT6 in root cells are enriched in genes bound by ZAT6 in planta (e.g. ChIP-seq; Figure 4C), there is no enrichment of the known ZAT6 cis-element in either set of ZAT6-regulated genes (induced or repressed; Figure 3C). Instead, the list of ZAT6-induced genes are specifically enriched is cis-elements for cis-clusters 6 and 39 from the bZIP/bHLH/BZR TF families (Figure 5C), whereas the list of ZAT6-repressed genes are enriched in cis-cluster 13 for WRKY TFs (*P* < 0.05, Fisher’s exact test; Figure 5C). These results suggest that ZAT6 regulates both its induced and repressed targets via interactions with members of these TF partner families.

When we analyzed all 14 focus TFs for potential partner TFs, we observed that cis-motif clusters 6 and 39 are enriched (*P* < 0.05, Fisher’s exact test) in the focus TF-induced and TF-bound gene target lists of 7/14 of the ABA-responsive TFs (Supplemental Table S8). Furthermore, we found that cis-motif Clusters 6 and 39 are enriched in the list of genes induced by ABA (*P* < 0.05, Fisher’s exact test), but not in the list of ABA-repressed genes (Supplemental Table S8). This result suggests that partner TFs from the bHLH/bZIP/BZR TF family/families work with MYB3, ZAT6, and other ABA-responsive focus TFs to regulate these ABA-responsive targets. Likewise, cis-motif cluster 13, which represents WRKY TFs, is enriched in the list of the focus TF-repressed and TF-bound targets of 7/14 TFs, as well as in the list of genes that are repressed in response to ABA (*P* < 0.05, Fisher’s exact test; Supplemental Table S8).

Overall, our cis-analyses using ConnecTF uncovered potential partner TFs for 14/21 focus TFs previously identified to be involved in the ABA response (Song et al., 2016).

### Case study 2: Refining/pruning inferred GRNs using validated TF–target data

In this case study, we show how ConnecTF can be used to readily combine and evaluate the relevance of gold-standard TF–target gene validation data to perform automated precision/recall analysis. Such results can be used to refine/prune TF–target connections in inferred GRNs. This feature will advance the systems biology cycle of network prediction, validation, and pruning/refinement.

#### Performing automated precision/recall analysis and refinement/pruning of a nitrogen-response GRN

**Figure 6:**
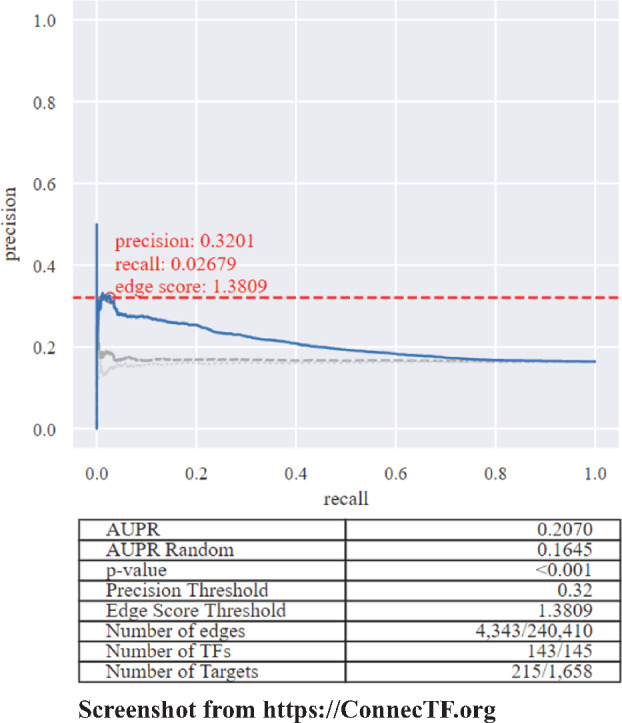
Case study 2: An automated precision/recall analysis performed on an inferred network uploaded to ConnecTF. Users are able to use functions in ConnecTF to perform an automated precision/recall analysis on a predicted/inferred GRN. To do this, the user first uploads a ranked list of TF–target interactions in a predicted network into ConnecTF from the *Query* page using the *Target Network* box. Next, users can validate/refine their predicted network using validated TF–target gene data housed in the ConnecTF database. Once they do this, within the *Network* tab, a precision/recall analysis (AUPR) section will be automatically generated for the predicted network, using selected TF–target validation datasets in the ConnecTF database, and displays a precision/recall plot and summary table. The user can then select a precision cutoff using the sliding bar above the plot, which will interactively update the AUPR graph, summary table, and the network that is visualized or exported. Query filters enable the user to select which TFs and the specific types of edges that should be used as the “gold standard” to perform precision/recall analysis of the predicted network. Here, we show a screenshot for an example where we used the time-based inferred network from Arabidopsis roots (Brooks et al., 2019), and all validated edges from TFs whose TF-regulated targets were identified in root cells using the TARGET assay (39 experiments) to demonstrate this AUPR feature of ConnecTF. *Query*: all_expression[TISSUE/SAMPLE="Root Protoplasts"], *Target Network*: Root_Nitrogen_Predicted_Network, *Background*: TARGET_Expressed.

As an example, we show how ConnecTF can automate a precision/recall analysis on a GRN inferred from time-series transcriptome data of the nitrogen response in Arabidopsis roots (Brooks et al., 2019). As gold-standard validation data, we selected the TF–target regulation data based on TF-perturbation experiments performed in root cells using the TARGET system (Bargmann et al., 2013). This set of 55 TFs includes the 33 nitrogen-responsive TFs from Brooks et al. (2019), 8 TFs that act downstream of the master nitrogen-response TF, NLP7 (Alvarez et al., 2020), and TARGET data for the 14 ABA-response TF–target regulation datasets generated in root cells in our current study (Supplemental Table S2). To initiate this precision/recall analysis of the inferred nitrogen-response GRN in ConnecTF, we first queried the 55 TF–target gene regulation datasets performed in root cells using the *Query* page. To determine which of these 55 TFs were relevant to our GRN analysis, we used the *Target Network* box to select the “Root Predicted Nitrogen Network” from Brooks et al. (2019). This query returned a total of 32/55 queried TFs and 1,349 validated TF–target gene interactions in the predicted nitrogen-response GRN. This query automatically generates a precision/recall curve, which is seen in the AUPR section at the bottom half of the *Network* tab (Figure 6). The slider or textbox above the precision–recall plot can be used to select a precision cut-off score, which will update the interactive graph and table with details of a pruned/refined network (e.g. the predicted TF–target interactions whose edge score equals or exceeds the selected precision score threshold). In this example, the selected TF–target edge score cutoff of 0.32, reduced the size of the predicted nitrogen-regulatory GRN from 240,410 interactions between 145 TFs and 1,658 targets, to a refined higher-confidence GRN. This higher confidence GRN is composed of 4,343 interactions between 143 TFs and 215 target genes whose predicted interactions passed the 0.32 threshold set by the precision/recall analysis of the validated TF–target gene interactions (Figure 6).

GRNs constructed based on co-expression data can also be validated in a similar manner. To this end, we provide a precision/recall example for a GRN built from the co-expression network available in the Atted-II database (Obayashi et al., 2018). We pruned this co-expression GRN using all TF-regulation data in the ConnecTF database (Supplemental Figure S2).

Using the appropriate buttons at the top of the *Network* page, the user can download the pruned/refined network as a network file (in JSON or SIF formats) or visualize the network in the browser (*Open Network*). The precision cutoff can be further modified while viewing the network in the browser using the slider or text box in the *Additional Edges* menu. TF–target edges within the network can be hidden to highlight a specific interaction type of interest (e.g. time-based TF–target edge predictions) or additional edges can be added from a file the user uploads. The resulting pruned network can be saved as a JSON file or an image exported.

#### TF-regulation data outperforms in vitro TF-binding data as a gold standard for precision/recall analysis

Next, we demonstrate how ConnecTF can be used to evaluate which TF–target validation datasets are most effective for use as gold standards for GRN refinement. As an example, the automated functions in ConnecTF enabled us to rapidly evaluate and compare the relative AUPR performance of different TF–target validated datasets (TF-binding vs. TF-regulation) in precision/recall analysis of a GRN inferred from time-series nitrogen response in Arabidopsis roots (Brooks et al., 2019). The TF–target validated datasets we tested are: (1) TF-regulated gene sets; TF–target genes regulated in root cells using the TARGET assay (Brooks et al., 2019; Alvarez et al., 2020), (2) TF-bound gene sets; TF–target sets bound in vitro (DAP-seq; O'Malley et al., 2016), or (3) Intersection of TF-regulated and TF-bound gene sets; TF–target sets regulated in root cells (TARGET assay) *and* bound in vitro (DAP-seq; Table 2). For the gene sets that involved TF–target binding (i.e. 2 and 3 above), we also used the DHS data (Sullivan et al., 2014) housed in the ConnecTF database to filter for DAP-seq peaks that occur in open chromatin regions in root tissue.

**Table 2:** Case study 2: Precision/recall analysis of a GRN inferred network from time-series nitrogen-response data in Arabidopsis roots (Brooks et al., 2019) performed using automated precision/recall functions in ConnecTF using different sets of experimentally validated edges in the ConnecTF database

Validated edges used	AUPR	AUPR randomized network	*P*-value	Percent improvement vs. random (%)
TF-regulated only (TARGET)	0.2025	0.1595	<0.001	27
TF-bound only (in vitro; DAP-seq)	0.3257	0.2967	<0.001	10
TF-regulated and TF-bound (in vitro; TARGET ∩ DAP-seq)	0.0863	0.0614	<0.001	41
TF-bound only (in vitro; DAP-seq)/ DHS filtered (root)	0.1908	0.1682	<0.001	13
TF-regulated and TF-bound (in vitro)/ DHS filtered (root; TARGET ∩ DAP-seq)	0.0555	0.0398	<0.001	39

By comparing the precision/recall results on networks refined using these three validated TF–target gene datasets, we found that using TF-regulated target data identified in root cells using TARGET as the “gold standard” resulted in a higher AUPR, and greater improvement in AUPR relative to the randomized predicted network, compared to using in vitro TF-binding target data alone (DAP-seq; Table 2). In addition, we found that whereas combining TF–target regulated and TF–target bound datasets reduced the AUPR, it also resulted in a greater improvement in the AUPR relative to the randomized network, compared to using TF-regulation datasets only. Finally, we found that applying the DHS filter to DAP-seq peaks reduced the AUPR, and only had a small effect on the improvement of the AUPR relative to the randomized network, compared to the same set of edges without the DHS filter (Table 2). Thus, the ability to test and combine TF–target datasets in an automated AUPR analysis enabled us to rapidly determine which of the tested datasets were the most effective for use in precision/recall analysis and network refinement.

### Case study 3: Charting a network path by combining validated TF–target data for multiple TFs

An important feature that distinguishes ConnecTF from most other available TF analysis tools/platforms is its *Query* building function. The *Query* builder allows users to readily select, parse, and combine TF–target gene validation data from different TF experiments and research groups stored in the ConnecTF database. For example, we demonstrate in the steps outlined below how ConnecTF can be used to chart a network path from the direct targets of a TF_1_ to its indirect targets via secondary TFs (TF_2_s). We initially conceived of this Network Walking approach which we manually executed in Brooks et al. (2019). As a working example, we show how ConnecTF can be used to chart a network path from a TF_1_ (e.g. NLP7, a master TF in the nitrogen signaling pathway) to its direct TF_1_–targets to its indirect targets. We did this by combining TF–target regulation and TF–target binding datasets from two different NLP7 studies: one performed by NLP7 perturbation in root cells and one performed in planta (Marchive et al., 2013; Alvarez et al., 2020).

#### Step 1. Identify direct vs. indirect targets of TF_1_

**Figure 7:**
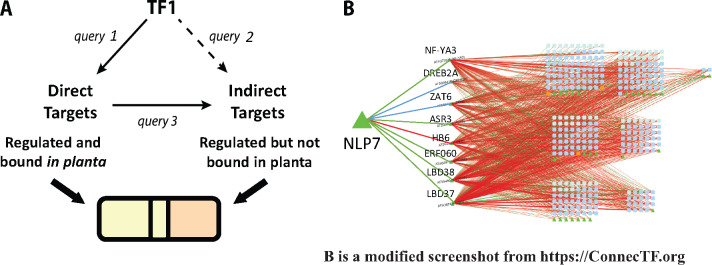
Case study 3: Network Walking: Using ConnecTF to chart a network path from TF_1_ → TF_2_s → indirect targets of TF_1_. The query system of ConnecTF can be used in an iterative process, with the results of one query being used to filter the TFs and/or target genes of other queries. This facilitates the building of more complex GRNs, such as charting a network path from TF_1_ to its downstream TF_2_s and indirect targets. A, ConnecTF can be used to chart a network path from a TF_1_ via its direct TF_2_s to its indirect targets using the Network Walking approach described in Brooks et al. (2019). Simple queries can be used in ConnecTF to integrate TF–target binding and TF–target regulation datasets to identify TF_1_ direct targets (TF_1_-regulated and TF_1_-bound, query 1) and TF_1_ indirect targets (TF_1_-regulated but not TF_1_-bound, query 2). The results of a query can also be saved and used to filter subsequent user queries, as in query 3. B, We demonstrate the process of Network walking using NLP7, a master TF_1_ involved in nitrogen signaling, identifying a set of eight direct intermediate TF_2_s targets acting downstream of NLP7 that control 68% of the NLP7 indirect targets. *Query 1*: AT4G24020[EXPERIMENT_TYPE=Expression] and AT4G24020[TECHNOLOGY/METHOD=ChIPseq], *Query 2*: AT4G24020[EXPERIMENT_TYPE=Expression] and not AT4G24020[TECHNOLOGY/METHOD=ChIPseq], *Query 3:* all_expression[TISSUE/SAMPLE="Root Protoplasts"], *Filter TFs*: Targets from query 1 e.g. Bound and regulated (direct) by NLP7, *Target Genes*: Targets from query 2 e.g. Regulated but unbound (indirect) by NLP7.

The first step in charting a network path is to identify the direct vs. indirect targets of TF_1_. To this end, we used the *Query* function in ConnecTF to identify direct NLP7 (TF_1_) targets as genes that are both NLP7-regulated and -bound (Marchive et al., 2013; Alvarez et al., 2020). Next, we identified indirect NLP7 targets as genes that are NLP7-regulated, but not bound by NLP7 in ChIP experiments (Marchive et al., 2013; Alvarez et al., 2020). We executed two simple queries in ConnecTF to produce these lists of direct targets of NLP7 (Figure 7A, Query 1) and indirect targets of NLP7 (Figure 7A, Query 2)*.* The list of genes resulting from these queries can be saved within ConnecTF, to be used as direct vs. indirect target gene lists of the TF_1_ (NLP7) for further analyses in the following steps, or downloaded by the user.

#### Step 2. Connect TF_1_ to its indirect targets via its direct intermediate TF_2_s

With the lists of direct vs. indirect targets of a TF_1_ (NLP7) in ConnecTF, we can now perform the second step of charting a network path in the Network Walking approach. In Step 2, we used ConnecTF to connect NLP7 to its indirect targets via TF_2_s that are themselves direct targets of NLP7. To do this, we queried the ConnecTF database for all the TF–target regulation datasets performed in root cells using TARGET (55 TFs). We further restricted the results returned to the indirect targets of TF_1_ (e.g. NLP7 regulated, but not bound) using the *Target Genes* filter on the query page. For this query, we also restricted the TF_2_s to the direct targets of NLP7, as identified in Step 1, using the *Filter TFs* option (Figure 7A, Query 3). The resulting *Table* tab shows the complete set of validated TF–target edges from eight TF_2s_ that are direct targets of NLP7 (e.g. TF_2_s: ASR3, NF-YA3, DREB2A, ZAT6, ERF060, HB6, LBD37, and LBD38) to NLP7 indirect targets. From the *Target Enrichment* tab, we see that all eight TF_2_s are enriched for NLP7 indirect targets (*P* < 0.05, Fisher’s exact test), with NF-YA3, LBD37, and LBD38 being the most important based on TF-influence, target specificity, and *P*-value of the overlap (Supplemental Figure S3).

#### Step 3. Visualizing the network path from TF_1_ → direct TF_2_(s) → indirect targets of TF_1_

Finally, we can visualize the resulting network path from TF_1_ (NLP7) → eight direct TF_2_ targets → indirect TF_1_ targets. We can do this in ConnecTF by going to the *Network* tab and clicking *Open Network*, which will launch Cytoscape.js (Franz et al., 2015). Basic Cytoscape functionality is available within ConnecTF for viewing and adding additional edges to the network (Figure 7B), or the network can be downloaded as a JSON file and further modified by the user.

## Discussion

Herein, we describe the development and deployment of ConnecTF (https://ConnecTF.org), a software platform designed to facilitate the integration of validated TF–target gene interaction datasets and harness them to create, refine, and prune GRNs; a current bottleneck in the systems biology cycle.

In case study 1, we used the functions in ConnecTF to perform an integrated analysis of TF-regulation data generated in our study (Supplemental Table S2) and TF-binding datasets for 14 of the TFs in the ABA-response pathway (Song et al., 2016). This integrated analysis enabled us to discover distinct TF modes-of-action for each focus TF and identify its putative partner TFs (Figure 5).

The simplest model for TF–target gene regulation is through direct interaction of a TF via DNA-binding to cis-regulatory regions in its target genes, and our results support that most TFs are able to regulate target gene expression in this way. We also uncovered evidence for indirect action of a focus TF on its targets via focus TF-partner TF cooperativity, which has been shown to play an essential role in how a TF controls target gene expression (Yáñez-Cuna et al., 2012; Para et al., 2014; Slattery et al., 2014; Alvarez et al., 2020; de Boer et al., 2020). Previous studies have identified putative TF partners by looking at co-occurrence of binding using ChIP peaks, as in the ENCODE and maize studies (Gerstein et al., 2012; Tu et al., 2020), and we use their focus TF and partner TF terminology in our analyses. By integrating TF-regulation and TF-binding data, we also found evidence that direct binding of a TF to a target could lead to gene activation, whereas indirect binding could lead to gene repression (or vice versa; Figure 5). Moreover, we showed how ConnecTF can be used to identify potential partner TFs involved in the indirect target binding of the focus TF. This was done by determining the enrichment of cis-binding motif clusters for partner TF families (Brooks et al., 2019) in the direct regulated targets of the focus TFs (Figure 5). In these cases, the results suggest that regulation could occur by indirect focus TF binding to a target gene via its association with partner TFs, sometimes referred to as “tethering” (Stender et al., 2010).

We also found that 3/14 TFs tested (ABF1, ABF3, and DREB2A) were able to repress the expression of a set of target genes with no evidence for direct or indirect binding to the gene targets (Supplemental Tables S5 and S7). Other regulatory mechanisms for transcriptional control have been reported that do not involve TF binding, either direct or indirect, to target genes. This includes the destabilizing of transcriptional complexes by a TF, as seen for SPL9 repression of anthocyanin biosynthesis (Gou et al., 2011), and TFs sequestering components of a transcriptional activating complex (Nemie-Feyissa et al., 2014). Overall, these results demonstrate how ConnecTF can be used to generate testable hypotheses by integrating TF-regulation and TF-binding datasets that can be used by the user for further investigation.

Our integration of TF-regulation and TF-binding studies also revealed that TF-regulation is a good indicator of TF-binding, but that TF-binding is a poor indicator of TF-regulation (Figure 4D). Specifically, for 14 TFs analyzed, up to 78% of the direct TF-regulated genes identified in root cells using TARGET were TF-bound in planta (Figure 4D and Supplemental Table S7). However, the reverse is not the case, as we find that at most 24% of TF–targets bound in planta were TF-regulated in root cells, for these 14 TFs (Figure 4D and Supplemental Table S7). Whereas this could be due to the different experimental design used in the studies being compared, TF-binding is well-known to be a poor indicator of TF-regulation across many eukaryotic organisms, even when TF-regulation and TF-binding are compared from the same tissue (Phuc Le et al., 2005; Gitter et al., 2009; Bolduc et al., 2012; Arenhart et al., 2014), or even from the same cell samples (Para et al., 2014).

In case study 2, we demonstrated how ConnecTF can be used to overcome the bottleneck in systems biology, which is the validation of predicted networks using TF–target validation data. We show how automated functions in ConnecTF allow a user to readily select and test gold-standard validated TF–target interactions to perform precision/recall analysis of GRNs (Figure 6). We used these automated precision/recall analysis features in ConnecTF to discover that TF–target regulation datasets outperform in vitro TF-binding datasets as gold-standard data in AUPR tests (Table 2). These results are unsurprising given that we observed in vitro binding (i.e. DAP-seq) is extensive in the genome, but often represents only a subset of TF-regulated targets, as shown in case study 1 (Figure 4D). This is likely related to the observation that a majority of TF-binding in the genome does not result in gene regulation (Supplemental Table S6), and/or TF–TF interactions (i.e. indirect binding), which are not captured in this in vitro DNA-binding assay.

In case study 3, we showed how the ConnecTF platform enables users to integrate validated TF–target gene interactions from multiple TF datasets into a unified network path within a GRN, facilitating systems biology studies. To demonstrate this, we used ConnecTF to chart a network path that defined how NLP7, a master regulator of nitrogen signaling (Marchive et al., 2013; Alvarez et al., 2020), controls downstream genes through intermediate TF_2_s, following the Network Walking approach developed previously (Brooks et al., 2019). To do this, we showed how simple queries in ConnecTF allowed us to identify eight direct TF_2_ targets of NLP7 that are able to directly regulate 68% of NLP7 indirect targets (Figure 7). This network path shows that LBD37 and LBD39, which are known to be important in nitrogen uptake and assimilation in planta (Rubin et al., 2009), are the TF_2_s that are the most influential on NLP7 indirect targets (Supplemental Figure S3). Thus, ConnecTF offers a user-friendly way to identify the sequential action of TFs in a network path that regulate a pathway or set of genes of interest.

## Conclusion

These three case studies are just a few examples of the many ways that ConnecTF will be able to facilitate the systems biology cycle of network generation, refinement, and validation across the plant community. Importantly, it is a user-friendly platform that will enable researchers to integrate the vast amount of diverse TF–target validation datasets to refine/prune inferred GRNs. We will host and maintain databases for the plant species Arabidopsis, maize, and rice. Importantly, we built the ConnecTF framework with common software packages and a species-independent structure. Thus, it is possible for users to easily set up an instance of ConnecTF for any species of interest, and/or add new features and analysis tools. We provide detailed instructions on how to build private and/or public versions of ConnecTF for users interested in creating a database with their own data, and encourage researchers to do so for their species of interest. As more TF-centric data is generated, we expect ConnecTF to be a powerful and easy-to-use tool to integrate validated interactions into transcriptional regulatory networks in plants and other species.

## Materials and methods

### Validation of TF-regulated targets in isolated plant cells

To identify the direct regulated targets of the 14 TFs in the ABA pathway that had both in planta ChIP (Song et al., 2016) and cis-binding motifs available (Weirauch et al., 2014), we expressed the TFs in isolated root cells using the TARGET system, using the transient expression vectors described in Brooks et al. (2019) as follows. Arabidopsis Col-0 plants were grown in 1% w/v sucrose, 0.5 g L^−1^ MES, 0.5× MS basal salts (–CN), 2% (w/v) agar, pH 5.7 for 10 d. Light conditions were 120 *μ*mol m^−2^ s^−1^ at constant temperature at 22°C, 16 h light, 8 h dark. Roots were harvested and stirred with cellulase and macerozyme (Yakult, Japan) for 3 h to remove the cell wall. Root protoplasts were filtered through 70-µm and then 40-µm cell strainers (BD Falcon, USA) and pelleted at 500*g*. Filtered root cells were washed with 15 mL MMg buffer (400-mM mannitol, 10-mM MgCl_2_, 4-mM MES pH 5.7) and resuspended to between 2–3 × 10^6^ cells per mL. Transfections of root cells with plasmid vectors described below were performed in a 50-mL conical tube by mixing 1 mL of root cell suspension with 120 *μ*g of plasmid DNA, 1 mL of PEG solution (40% (w/v) polyethylene glycol 4000 (Millipore Sigma, USA), 400-mM mannitol, and 50-mM CaCl_2_) and vortexed gently for 5 s. After mixing, 50 mL of W5 buffer (154-mM NaCl, 125-mM CaCl_2_, 5-mM KCl, 5-mM MES, 5-mM glucose, pH 5.7) was added to the tube. Root cells were pelleted at 1,200*g*, and washed three times with W5 buffer. Cells transfected with either an empty vector (EV) as a control, or a single TF cloned in the pBOB11 plasmid vector containing an RFP selectable marker (pBOB11, available at https://gatewayvectors.vib.be/collection (Bargmann et al., 2013)) and another batch of cells were transfected with a single TF in the pBOB11-GFP plasmid vector (pBOB11-GFP, available at https://gatewayvectors.vib.be/collection (Brooks et al., 2019)) were aliquoted into three replicate wells of a 24-well plate. The following day (18 h) after TF expression and translation, transfected root protoplasts were treated with 35-*µ*M CHX for 20 min before a 10-*µ*M DEX treatment to induce TF nuclear import. Transfected root cells expressing each TF or EV were sorted into GFP and RFP-expressing root cell populations by FACS 3 h after DEX treatment.

To identify TF-regulated genes, transcriptome analysis was performed. For this, cells expressing the candidate TF vs. EV were collected in triplicate and RNA-Seq libraries were prepared from their mRNA using the NEBNext^®^ Ultra™ RNA Library Prep Kit for Illumina^®^. The RNA-Seq libraries were pooled and sequenced on the Illumina NextSeq 500 platform. The RNA-Seq reads were aligned to the TAIR10 genome assembly using HISAT2 (Kim et al., 2019) and gene expression estimated using the GenomicFeatures/GenomicAlignments packages (Lawrence et al., 2013). Gene count tables were combined for each TF sample and the EV and differentially expressed genes in the TF transfected samples vs. the EV samples were identified using the DESeq2 package (Love et al., 2014) with a TF+Batch model and an FDR adjusted *P* < 0.05. We filtered out genes that respond more than 5-fold to CHX treatment in EV transfected protoplasts (Brooks et al., 2019) from the lists of TF-regulated gene targets. Genes that are expressed in any of the TF- or EV-transfected protoplast experiments were used as the background for subsequent enrichment analyses in ConnecTF (Supplemental Table S9).

### Reanalysis of rice TF–target datasets

NCBI GEO and SRA databases were queried for sequencing data related to TF perturbations of any rice TFs. We examined the ChIP-seq data and RNA-seq data for TF overexpression and loss-of-function lines. TF-regulated genes identified by differential expression (RNA-seq) and TF-bound genes (ChIP-seq) in supplemental files from published papers were added to ConnecTF only if the list was comprehensive (e.g. for the whole genome) and if the dataset included more than one replicate for each condition. If this information was not provided, we re-processed the raw sequencing reads from SRA and aligned them to the MSU7 genome (Kawahara et al., 2013). For RNA-seq, raw sequences were trimmed by fastp 0.19.10 (Chen et al., 2018) and aligned to the genome using STAR 2.5.3a (Dobin et al., 2012). The gene counts matrices were generated by featureCounts 1.6.3 (Liao et al., 2013) and differentially expressed genes were called with DESeq2 (Love et al., 2014) with a FDR < 0.05. For ChIP-seq, raw sequences were trimmed by fastp 0.19.10 and aligned to the genome using Bowtie2 2.3.4 (Langmead and Salzberg, 2012). Duplicated reads were removed by samblaster 0.1.24 (Faust and Hall, 2014). Reads with MAPQ score lower than 10 were removed by samtools 1.9 (Li et al., 2009). MACS2 (Zhang et al., 2008) was used to call peaks and bedtools closest (Quinlan and Hall, 2010) was used to identify bound genes (within 2 kb upstream and downstream). If ChIP-seq peaks files were provided by the authors, we used those to identify TF-bound genes.

### TF–target list enrichment

Target list enrichment calculates the significance of the overlap between TF–targets in each queried TF analysis and each user-uploaded gene list. The *P*-values are calculated using the Fisher’s exact test adjusted with the Bonferroni correction. The background set of genes used for the calculation, which is by default all protein-coding genes for the Arabidopsis, rice (*Oryza sativa*) and maize (*Zea mays*) instances of ConnecTF, can be manually set by the user by using the *Background Genes* option in the query page.

### cis-motif enrichment

Arabidopsis, rice, and maize cis-binding motif PWMs were collected from Cis-BP (Weirauch et al., 2014; Build 2.0) and the 80 cis-motif clusters of Arabidopsis were obtained from Brooks et al. (2019) and converted to MEME format. The FIMO tool (Grant et al., 2011) within the MEME package (Bailey et al., 2009) was used to identify every occurrence of each cis-binding motif in the nuclear genome (i.e. excluding mitochondrial and chloroplast chromosomes) at a *P* < 0.0001 using the base frequency in the nuclear genome as the background model.

We chose to remove overlapping sites for the same cis-binding motifs, which are particularly common for repetitive motifs. For each cis-binding motif, when two sites overlap, the match with the lowest *P*-value is kept, and the other is removed until only non-overlapping matches remain. The number of matches for each cis-binding motif is tallied for each individual gene region, subdivided into 2,000, 1,000, and 500 bp upstream of transcription start site, the 5’ and 3’ UTRs, CDS, intron, exon, and the full region transcribed into mRNA (cDNA). If a match is found to be within a region shared by more than one gene, it is counted for all the genes that it is associated with.

To calculate enrichment of a cis-binding motif or cis-motif cluster for a particular individual TF within a given region in a target gene of a queried analysis, the Fisher’s exact test was used with a background of all individual cis-binding motifs or cis-motif clusters within that gene region, respectively. As in *Target List Enrichment*, a user can upload a list of genes to use as the background, or use the default of all protein-coding genes. The *P*-values are adjusted with the Bonferroni correction method.

If a target gene list (e.g. genes in a pathway of interest) is provided by the user, ConnecTF can also calculate the cis-binding motif enrichment for that gene list(s), separately. The *P*-values of motif enrichment on gene lists is adjusted with the Bonferroni correction as a group, independent of the correction performed on the queried analyses.

### Gene set enrichment

The gene set enrichment tool (Katari et al., 2010; Krouk et al., 2010) calculates the significance of overlap between all possible pairwise combinations of target gene lists identified for any TF–targets queried. Significance of overlap is calculated using the one-sided Fisher’s exact test, using the default background of all protein coding genes, or the user uploaded background. On the resulting grid, cells above the diagonal report the *P*-value for the upper tail (greater or equal to the observed overlap) and cells below the diagonal report the *P*-value for the lower tail (lesser or equal to the observed overlap). All the *P*-values are adjusted with the Bonferroni correction.

### Sungear, a visualization method for gene set overlaps

Sungear (Poultney et al., 2006) is a tool to display/visual overlaps between gene lists resulting from different queries, similar to a Venn diagram or UpSet plot (Lex et al., 2014). The vertices on the outer polygon are anchor points, containing gene lists for each TF-analysis queried. Circular nodes within the polygon represent gene sets that are unique to or in common between the indicated lists of genes in the vertices, based on their position between the vertices. Each node has one or more arrows pointing to the vertices corresponding to the analyses that contain the genes. The gene sets exclusively found in that node represents the specific combination of analyses. The position of the node is approximately the midway point between the combination of analyses it represents.

In our implementation of Sungear within ConnecTF, we enhanced the graph by calculating a *P*-value, which indicates whether a node contains greater or fewer overlap of genes than expected given the total number of targets regulated by each of the queried analyses. Calculation was performed using the following method.

Let us say there are n lists, each containing *x*_1_, *x*_2_ … *x_n_* number of genes, with a total of *x* genes.

$$x=\sum_{i=1}^{n}{\;x}_{i}$$

If a node $A_{1,2,4}$ indicates genes that are exclusively in common with lists 1, 2, and 4. Then the expectation value, e, of a gene being in that node can be calculated from multiplying probability of being in the gene list and not being in the gene list respectively and *x*.

$$e_{A_{1,2,4}}=\left(\frac{x_{1}}{x}\right)\cdot \left(\frac{x_{2}}{x}\right)\cdot \left(\frac{x_{4}}{x}\right)\cdot \left(1-\;\frac{{\;x}_{3}}{x}\right)\cdot \left(1-\;\frac{{\;x}_{5}}{x}\right)\cdot \ldots \cdot \left(1-\;\frac{{\;x}_{n}}{x}\right)x$$

This will be a binomial distribution, where success is defined as the number of genes in the node A, and the failure is the number of genes not in node A (total genes − number of genes in node A). The *P*-value is calculated for each node by comparing the observed value to the expected value using the binomial test and adjusted using the Bonferroni correction.

## Code availability

The source code including instructions for setting up a public or private instance of ConnecTF is available at https://github.com/coruzzilab/connectf_server.

## Accession numbers

All raw sequencing data from this project have been deposited in the Gene Expression Omnibus (GEO) database, https://www.ncbi.nlm.nih.gov/geo (accession no. GSE152405).

## Supplemental data

The following materials are available in the online version of this article.

**Supplemental Figure S1.** Diagram of ConnecTF from data to analysis tools.

**Supplemental Figure S2.** Case study 2: Precision/recall analysis on the Atted-II co-expression network.

**Supplemental Figure S3.** Case study 3: Enrichment of TF_2_ targets with NLP7 indirect targets reveals influential downstream TFs.

**Supplemental Data File.** ConnecTF queries used to generate figures and tables.

**Supplemental Table S1.** Overview of the data in the maize and rice instances of ConnecTF.

**Supplemental Table S2.** Direct regulated targets of 14 ABA-responsive TFs identified using the TARGET system in root cells.

**Supplemental Table S3.** Table of results for NLP7 targets collected from each of the NLP7 experiments in ConnecTF.

**Supplemental Table S4.** Case study 1: Enrichment of ABA-responsive genes in the induced or repressed direct regulated TF-regulated targets.

**Supplemental Table S5.** Case study 1: Motif enrichment in induced or repressed direct regulated TF targets.

**Supplemental Table S6.** Case study 1: Genes set enrichment of induced or repressed direct regulated TF targets in cells with in vitro TF-bound targets (DAP-seq).

**Supplemental Table S7.** Case study 1: Genes set enrichment of induced or repressed direct regulated TF targets in cells with in vivo TF-bound targets (ChIP-seq).

**Supplemental Table S8.** Case study 1: cis-Motif cluster enrichment in induced and bound or repressed and bound TF–targets.

**Supplemental Table S9.** List of genes that are expressed in TARGET experiments and used as background for enrichment analyses.

## Supplementary Material

kiaa012_Supplementary_Data

## Notes

M.D.B., C.-L.J., and M.S.K. designed the database and website, with input on content and functionalities from G.M.C.; C.-L.J. built the database and website; A.P. performed the TARGET TF-validation experiments and H.-J.S. created the RNA-seq libraries; M.D.B. and J.M.A. analyzed the RNA-seq data; J.H. and C.S. analyzed additional published datasets; J.C. contributed to the development of analysis tools; M.D.B. and G.M.C. conceived the project and wrote the article, which all the authors revised.

The author responsible for distribution of materials integral to the findings presented in this article in accordance with the policy described in the Instructions for Authors (https://academic.oup.com/plphys) is: G.M.C. (gloria.coruzzi@nyu.edu).
