# Supplementary figures and images for: ConnecTF: A platform to integrate transcription factor–gene interactions and validate regulatory networks
Source: Plant Physiol. 2020 Nov 18;185(1):49–66. doi: 10.1093/plphys/kiaa012 (PMC8133578; doi:10.1093/plphys/kiaa012)

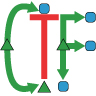

Supplement: kiaa012_Supplementary_Data [file kiaa012_supplementary_data.zip › kiaa012-suppl_data/pp.00899.2020-s03.jpg]
